# Multifunctional Sandwich‐Structured Electrolyte for High‐Performance Lithium–Sulfur Batteries

**DOI:** 10.1002/advs.201700503

**Published:** 2018-01-02

**Authors:** Hongtao Qu, Jianjun Zhang, Aobing Du, Bingbing Chen, Jingchao Chai, Nan Xue, Longlong Wang, Lixin Qiao, Chen Wang, Xiao Zang, Jinfeng Yang, Xiaogang Wang, Guanglei Cui

**Affiliations:** ^1^ Qingdao Industrial Energy Storage Technology Institute Qingdao Institute of Bioenergy and Bioprocess Technology Chinese Academy of Sciences Qingdao 266101 P. R. China; ^2^ University of Chinese Academy of Sciences No.19A Yuquan Road Beijing 100049 China

**Keywords:** cellulose nonwoven materials, lithium–sulfur batteries, multifunctional materials, nanocarbon black, sandwich‐structured electrolytes

## Abstract

Due to its high theoretical energy density (2600 Wh kg^−1^), low cost, and environmental benignity, the lithium–sulfur (Li‐S) battery is attracting strong interest among the various electrochemical energy storage systems. However, its practical application is seriously hampered by the so‐called shuttle effect of the highly soluble polysulfides. Herein, a novel design of multifunctional sandwich‐structured polymer electrolyte (polymer/cellulose nonwoven/nanocarbon) for high‐performance Li‐S batteries is demonstrated. It is verified that Li‐S battery with this sandwich‐structured polymer electrolyte delivers excellent cycling stability (only 0.039% capacity decay cycle^−1^ on average exceeding 1500 cycles at 0.5 C) and rate capability (with a reversible capacity of 594 mA h g^−1^ at 4 C). These electrochemical performances are attributed to the synergistic effect of each layer in this unique sandwich‐structured polymer electrolyte including steady lithium stripping/plating, strong polysulfide absorption ability, and increased redox reaction sites. More importantly, even with high sulfur loading of 4.9 mg cm^−2^, Li‐S battery with this sandwich‐structured polymer electrolyte can deliver high initial areal capacity of 5.1 mA h cm^−2^. This demonstrated strategy here may open up a new era of designing hierarchical structured polymer electrolytes for high‐performance Li‐S batteries.

## 1 Introduction

The conventional Li‐ion batteries (LIBs) with theoretical gravimetric energy density lower than 400 W h kg^−1^ cannot well satisfy the ever‐growing demand of high energy storage systems, especially in the field of large‐scale electric transportation tools and renewable stationary energy storage systems.^1^, ^2^, ^3^ Therefore, there is a pressing need to exploit alternative energy storage systems with higher energy density. Lithium–sulfur (Li‐S) battery has attracted much attention due to its high theoretical gravimetric energy density up to 2600 Wh kg^−1^, which is 3–5 times higher than LIBs.^4^, ^5^, ^6^, ^7^ In addition, sulfur is naturally abundant, inexpensive, and environmentally friendly. However, the practical application of Li‐S battery is plagued by several problems: (1) the intrinsic poor ionic/electronic conductivity of both element sulfur (S) and discharge products (i.e., Li_2_S_2_, Li_2_S), causing low sulfur utilization; (2) large volume change between S and lithiation product Li_2_S can damage cathode structure, resulting in poor contact between active material and conductive matrix;^8^, ^9^ (3) the severe “shuttle effect” of high soluble long‐chain lithium polysulfides (Li_2_S*_n_* 4 ≤ *n* ≤ 8).^10^, ^11^ The item (3) is the biggest roadblock blocking the commercialization process of Li‐S battery. Specifically, long‐chain lithium polysulfides formed by the reduction of sulfur at the cathode side can migrate to the anode side and are chemically reduced on Li metal. Then, some of the reduced lithium polysulfides diffuse back to the cathode side and are reoxidized to polysulfides with various valences. The so‐called “shuttle effect” of polysulfides has detrimental effect on Li metal anode that causes severe self‐discharge phenomenon and shortened lifetime.^12^, ^13^, ^14^

To address these problems, great efforts have been paid on designing various nanostructured conductive host materials for nonconductive sulfur, such as carbon nanotubes/nanosphere,^15^ graphene,^16^ metal oxides/sulfides nanoparticles,^17^ and heteroatom‐doped carbon.^18^, ^19^, ^20^ The nanostructured conductive host materials can not only improve the electronic conductivity of cathode but also confine sulfur in the cathode side physically or chemically. However, large‐scale synthesis of these nanostructured cathodes with well‐defined morphology is complicated and expensive. In addition, the advanced nanostructured cathodes can only trap the polysulfides and improve the battery performances for a short term. The continuous capacity deterioration upon long‐term cycling indicates that the dissolution and diffusion of polysulfides are still existing.^21^ Recently, some new approaches, including multifunctional interlayers, modification of commercial separators and solid electrolytes, are developed to suppress the polysulfides shuttles. Among these methods, all‐solid‐state electrolytes are thought to be the most effective way to solve the foregoing issues due to its polysulfide‐impermeability in combination with some additional advantages such as lithium dendrite suppression and enhanced safety.^22^, ^23^ Unfortunately, the previously reported Li‐S battery based on solid electrolytes delivered unsatisfied battery performance at room temperature owing to the limited ionic conductivities and large electrode/solid electrolyte interfacial resistances.^24^, ^25^, ^26^ In comparison, gel polymer electrolyte is a promising candidate for the improvement of Li‐S battery.^27^, ^28^, ^29^, ^30^, ^31^, ^32^ Kang's group developed pentaerythritol tetraacrylate (PETEA)‐based gel polymer via in situ polymerization for Li‐S battery. The electrochemical performance has been enhanced to a certain extent. However, excess polysulfides between cathode and polymer electrolyte cannot be further utilized, which results in continuous capacity decay.

In this work, we proposed a sandwich‐structured nanocarbon black/cellulose nonwoven/poly(ethylene glycol)‐*block*‐poly(propylene glycol)‐*block*‐poly(ethylene glycol) (PEG‐PPG‐PEG) gel polymer electrolyte (hereafter abbreviated as “NCP‐CPE”) for Li‐S batteries. Both the cycle life and rate capability have been extremely improved. Cellulose is one of the most abundant, renewable resources on the earth and possesses outstanding properties such as biocompatibility, desired chemical stability, and environmental benignity.^33^, ^34^, ^35^, ^36^, ^37^ In addition, its abundant oxygen‐containing functional groups, such as hydroxyls, are efficient sulfur and polysulfide immobilizers, and thus cellulose is chosen as the supporting framework.^38^, ^39^, ^40^, ^41^ Furthermore, the carbon coating on cellulose nonwoven can physically block the migration of polysulfides, and functions as an upper collector which can provide more redox reaction sites to enhance the utilization of sulfur. PEG‐PPG‐PEG is an amphiphilic triblock copolymer, where the PEG segment is hydrophilic and PPG segment is hydrophobic. They can self‐assemble into micelles above their critical micelle concentration or above their critical micellization temperature which can increase toughness and viscosity of the polymer. Because of the aforementioned versatile merits, PEG‐PPG‐PEG has been used in various fields such as aggregation, detergency, dispersion stabilization, foaming, emulsification, lubrication, and rechargeable batteries.^42^ In our work, the PEG‐PPG‐PEG is coated on the anode side of cellulose which can enable uniform lithium stripping/plating and then improve Coulombic efficiency of the battery. It is demonstrated that Li‐S battery based on this sandwich‐structured polymer electrolyte displays extraordinary cycle stability for 1500 cycles and outstanding rate capability (with a reversible capacity of 594 mA h g^−1^ at 4 C). No doubt, this research will open a new avenue for designing and exploiting sandwich‐structured polymer electrolyte for high‐performance Li‐S battery.

## 2 Results and Discussion

### 2.1 Morphology Property

**Figure 1:**
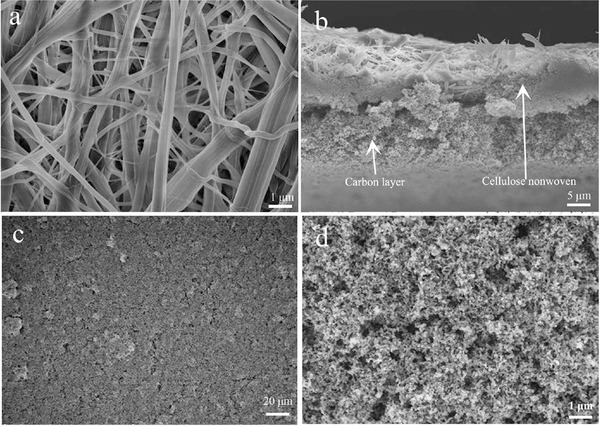
a) Typical SEM images of cellulose nonwoven. b) Cross‐section SEM image of NCP‐CPE. c) Top‐view SEM image of NCP‐CPE (500×) and d) NCP‐CPE (10000×).

Typical scanning electron microscopy (SEM) image of pristine cellulose membrane is shown in **Figure** **1**a. It can be seen that the cellulose nonwoven is consisted of randomly arranged fibers with diameters about 0.2–1 µm, which are different from PP separator with uniform and typically elliptic pores. The cellulose nonwoven with the tortuous pores formed during the papermaking process is in favor of enhancing the electrolyte uptake, which can significantly improve electrochemical performance of battery.

The cross‐section image of NCP‐CPE is presented in Figure 1b. It can be seen that the thickness of NCP‐CPE is ≈30 µm and the nanocarbon layer is about 15 µm. However, it is hard to see the polymer coating layer in the sandwich structure probably because the polymer immerses into the cellulose fibers during the preparation of polymer coating layer via the solution‐casting method. The transparent PEG‐PPG‐PEG solution would wet the cellulose nonwoven immediately and inosculate as a whole. As a result, it is difficult to distinguish the polymer coating layer from the cross‐section image of NCP‐CPE. The low‐magnification SEM image in Figure 1c indicates the full coverage of carbon coating on the surface of cellulose nonwoven. Figure 1d displays the high‐magnification surface morphology of NCP‐CPE. It can be clearly seen that an even carbon coating comprises close‐packed carbon nanoparticles which are interconnected by polyvinylidene fluoride (PVDF) binder. The nanosized carbon layer, homogeneously distributed on cellulose with significant pores, is favorable to enlarge the contact area between active sulfur species and conductive carbon nanoparticles. Moreover, the high conductivity of nanocarbon can also facilitate the electron transfer and enhance the electrochemical kinetics.^43^

### 2.2 Electrochemical Stability and Ionic Conductivity

**Figure 2:**
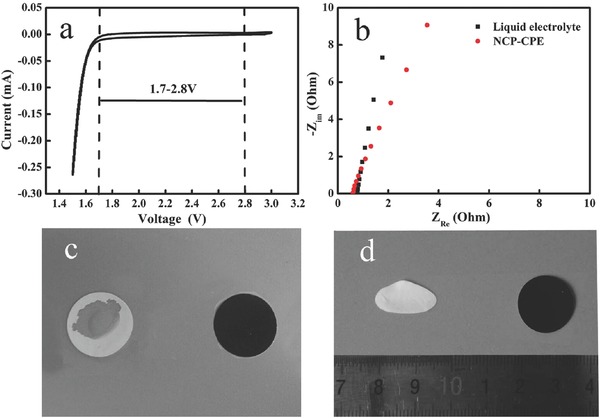
a) Cyclic voltammetry curves of Li/NCP‐CPE/SS cell. b) Electrochemical impedance plots of symmetric SS/NCP‐CPE/SS cell. c) Photograph showing liquid electrolyte wettability of PP separator and NCP‐CPE. d) Thermal shrinkage photographs of PP separator and NCP‐CPE after exposure to 150 °C for 0.5 h.

In order to investigate the electrochemical stability of the NCP‐CPE, cyclic voltammetry (CV) measurement was performed on a coin cell (using stainless steel (SS) as the working electrode and Li foil as the counter and reference electrode). In **Figure** **2**a, it is evident that there are no redox peaks during the CV measurement in the typical operating voltage range of Li‐S battery, indicating the NCP‐CPE can maintain good electrochemical stability during the charge–discharge process. As shown in Figure 2b, the bulk resistances of liquid electrolyte and NCP‐CPE are 0.78 and 0.60 Ω, respectively. The corresponding ion conductivity of liquid electrolyte and NCP‐CPE are calculated to be 1.8 × 10^−3^ and 2.3 × 10^−3^ S cm^−1^, respectively. The higher ion conductivity of NCP‐CPE can be attributed to the enhanced electrolyte uptake as shown in Figure 2c. It can be seen that the wettability of PP separator with electrolyte is poor, probably owing to its hydrophobic surface characteristic. In contrast, the cellulose‐based NCP‐CPE can be rapidly wetted by the liquid electrolyte, indicative of better affinity between the NCP‐CPE and the liquid electrolyte. This excellent wettability is relevant to the unique chemical structure of the cellulose and the porous structure of the nonwoven, which will facilitate the electrolyte fully filling in battery to achieve enhanced ion conductivity, which is extremely important for the Li‐S batteries.

In addition, highly dimensional stability of separator and electrolyte at elevated temperature plays an important role in the safety of batteries, for they can prevent short‐circuit by keeping anode and cathode apart even in harsh conditions. As shown in Figure 2d, NCP‐CPE maintains its original dimension even after thermal treatment at 150 °C for 0.5 h, while the traditional PP separator suffers from severe thermal shrinkage (>50%). The superior thermal tolerance makes NCP‐CPE very promising for thermally stable batteries. It is known to all that high mechanical strength is another pivotal parameter for the practical application of polymer electrolyte. Figure S2 (Supporting Information) shows the stress–strain curve of the NCP‐CPE. The maximum stress of NCP‐CPE is up to 28 MPa, which is sufficient for avoiding the internal short‐circuit of the batteries.

Generally, there are ample oxygen‐containing groups in cellulose. The functional groups in the cellulose are analyzed by Fourier transform infrared (FTIR) spectroscopy. As shown in Figure S3 (Supporting Information), the peak around 1035 cm^−1^ belongs to the (C—O) stretching modes of hydroxyl. The absorbance peak at 1315 cm^−1^ is assigned to the C—O groups of the aromatic ring in polysaccharides. An obvious broad peak at 3310 cm^−1^ is attributed to the hydroxyl group (OH) in cellulose.^44^

### 2.3 Charge/Discharge Curves, Rate Capability, and Cycle Performance of Lithium–Sulfur Batteries with 60% Sulfur Content

**Figure 3:**
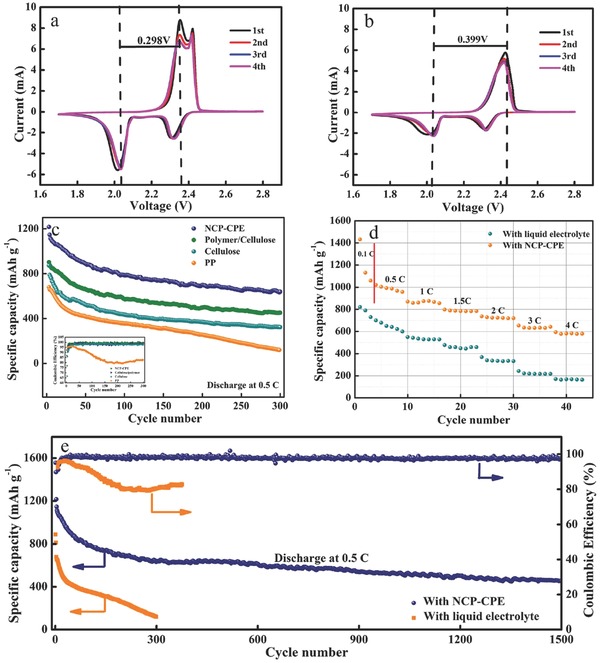
Cyclic voltammetry curves of Li‐S battery with a) NCP‐CPE and b) liquid electrolyte at a scan rate of 0.2 mV s^−1^. c) Cycle performance comparison of Li‐S battery with PP, cellulose, polymer/cellulose, and NCP‐CPE. d) Rate capabilities of Li‐S battery with liquid electrolyte and NCP‐CPE. e) Cycle performance comparison (0.5 C) of Li‐S battery using liquid electrolyte and NCP‐CPE.

CV results of Li‐S battery with NCP‐CPE are displayed in **Figure** **3**a. Two typical cathodic peaks can be distinctly found at 2.31 and 2.03 V (vs Li/Li^+^), corresponding to the transformation of S_8_ to soluble long‐chain polysulfides (Li_2_S*_n_* 4 ≤ *n* ≤ 8) and further reduction to insoluble short‐chain polysulfides (Li_2_S_2_/Li_2_S), respectively. During the reverse scanning process, the anodic peak located at around 2.38 and 2.4 V can be ascribed to the formation of long‐chain polysulfides and eventually element sulfur (S_8_). It is clear that all the subsequent cycles of CV overlap, demonstrating that the Li‐S battery with NCP‐CPE has outstanding electrochemical stability. As displayed in Figure 3b, typical cathodic and anodic peaks of Li‐S batteries are easily found when using routine liquid electrolyte. However, it is noteworthy that the battery with NCP‐CPE has a smaller potential gap (0.298 V) between the main cathodic and anodic peak than that (0.399 V) of Li‐S battery using liquid electrolyte. In addition, battery with NCP‐CPE exhibits much sharper current peaks, reflecting high conductivity and fast electrochemical kinetics.^28^

Figure 3c presents the cycle performance comparisons of Li‐S batteries with PP, cellulose nonwoven, polymer/cellulose, and NCP‐CPE at 0.5 C, respectively. Compared to the battery using PP separator, the battery with cellulose nonwoven shows higher capacity retention and Coulombic efficiency owing to the strong affinities between polysulfides and cellulose backbone. As expected, when the cellulose is coated with polymer, the Coulombic efficiency is improved (inset in Figure 3c), which is attributed to the steady lithium stripping/plating enabled by the polymer coating. It is obvious that the battery with NCP‐CPE shows the best performance within expectation. It can still deliver a satisfactory reversible capacity of 650 mA h g^−1^ after 300 cycles. The galvanostatic discharge/charge voltage profiles of Li‐S battery with liquid electrolyte and NCP‐CPE are presented in Figure S4 (Supporting Information). Obviously, both batteries show two typical discharge plateaus, which are consistent well with the corresponding CV curves in Figure 3a,b. As noted by the red arrow, Li‐S battery with NCP‐CPE delivers an overpotential of 110 mV, which is lower than that (160 mV) of battery with liquid electrolyte, indicating that battery with NCP‐CPE owns a lower polarization in the charge–discharge process, suggesting a kinetically efficient reaction process with a small barrier.^41^, ^42^ The enhanced performance is due to synergistic effect of each layer in the sandwich structure. The sulfophilic cellulose backbone can capture the dissolved polysulfides. With the assistance of carbon coating on cellulose backbone, the polysulfides adsorbed on cellulose get more opportunities to receive electrons from the current collector and then participate in the redox reactions, which will significantly improve the utilization of active sulfur species. Moreover, the polymer coating on the anode side of cellulose backbone can enable uniform lithium‐ion stripping/plating which can suppress the growth of dendrite.

The rate capabilities of battery with liquid electrolyte and NCP‐CPE are displayed in Figure 3d. As can be seen, a high initial capacity of 1429 mA h g^−1^ is obtained at 0.1 C. When the current density increase from 0.5 to 4 C, the battery delivers impressive discharge capacities of 1131, 969, 784, 729, 641, and 594 mA h g^−1^ at 0.5, 1, 1.5, 2, 3, and 4 C, respectively. In sharp contrast, the rate capability of control Li‐S battery is quite poor. The initial capacity is only 820 mA h g^−1^ at 0.1 C. When the rate increases to 4 C, a reversible capacity of 165 mA h g^−1^ is obtained.

Figure 3e shows the long‐term cycling behavior of Li‐S battery with liquid electrolyte and NCP‐CPE at 0.5 C. A remarkable improvement is achieved for Li‐S battery using NCP‐CPE. It delivers an initial capacity of 1146 mA h g^−1^ at 0.5 C. After continuous cycling for 1500 cycles, a satisfactory reversible capacity of 460 mA h g^−1^ is still obtained with an average capacity decay of only 0.039% cycle^−1^. By contrast, the Li‐S battery with liquid electrolyte gets an initial capacity of only 679 mA h g^−1^, and drastically drops to 100 mA h g^−1^ after 300 cycles. The Coulombic efficiency also sharply decreases to 80.8% after the first 100 cycles.

In addition, even at a high current density of 1 C, Li‐S battery with NCP‐CPE also exhibits good cycling stability for 400 cycles (Figure S5, Supporting Information). The battery exhibits an initial discharge capacity of 929 mA h g^−1^ and keeps at 601 mA h g^−1^ after 400 cycles. There is only a capacity decay of 0.047% cycle^−1^ on average. As summarized in Table S1 (Supporting Information),^45^, ^46^, ^49^, ^50^, ^51^, ^52^, ^53^, ^54^, ^56^, ^57^, ^58^, ^59^, ^60^, ^61^, ^62^, ^63^ Li‐S battery using NCP‐CPE shows a quite stable cycling performance for 1500 cycles and exhibits a superior rate capability up to 4 C with, which are comparable to the best reported literatures so far. The enhanced electrochemical performance of Li‐S battery is attributed to the following reasons: (1) carbon coating serves as an upper collector that provides more redox reaction sites; (2) the abundant hydroxyl groups in NCP‐CPE can effectively absorb the dissolved polysulfides, which significantly suppress polysulfides migration from cathode side to anode side; (3) steady lithium stripping/plating, which is attributed to PEG‐PPG‐PEG coating layer.^47^, ^48^

### 2.4 Cycle Performance of Lithium–Sulfur Batteries with 70 and 80% Sulfur Content

High sulfur content is essential for the implementation of high‐performance Li‐S battery. In the most published papers, the sulfur content is normally less than 65 wt%.^64^, ^65^ While in this paper, the sulfur content is highly increased to 70 and 80%. Figure S6a (Supporting Information) shows the cycle performance of Li‐S battery with 70% sulfur content. By employing NCP‐CPE, the battery delivers an initial discharge capacity of 929 mA h g^−1^ and retains at 717 mA h g^−1^ after 200 cycles. When the current density increases to 2 C (Figure S6b, Supporting Information), the battery obtains a high initial capacity of 867 mA h g^−1^ and maintains at 700 mA h g^−1^ after 200 cycles. As depicted in Figure S7a (Supporting Information), the battery with 80% sulfur is tested at 0.5 C and it can deliver a reversible capacity of 600 mA h g^−1^ for 300 cycles. Even cycled at 1 C (shown in Figure S7b, Supporting Information), a fabulous initial capacity of 631 mA h g^−1^ is still obtained. After 300 cycles, a reversible capacity of 405 mA h g^−1^ is obtained with a capacity decay of 0.119% cycle^−1^ on average. This enhanced electrochemical performance should be ascribed to the excellent polysulfide‐trapping ability of the sandwich‐structured NCP‐CPE.

High sulfur mass loading is also a key parameter for realizing high energy density Li‐S battery. Here, we also prepared a high sulfur loading cathode with mass density of 4.9 mg cm^−2^ (70% sulfur content). As shown in Figure S8 (Supporting Information), the battery using NCP‐CPE delivers a high initial discharge areal capacity of 5.1 mA h cm^−2^ at a current density of 0.34 mA cm^−2^. In addition, at a high current density of 0.79 mA cm^−2^, high sulfur loading Li‐S battery can still achieve a relatively good cycling stability for 50 cycles with reversible areal capacity of 3.5 mA h cm^−2^. These results suggest that NCP‐CPE can effectively mitigate the polysulfides shuttles and then dramatically increase the utilization of active sulfur‐related species.

### 2.5 Multifunctional Mechanism of Sandwich‐Structured Polymer Electrolyte in Li‐S Battery

**Figure 4:**
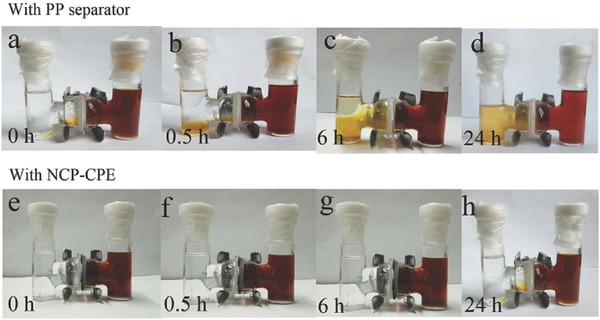
Digital photographs of H‐type cells separated by a–d) PP separator and e–h) NCP‐CPE.

In an attempt to visually demonstrate the NCP‐CPE's capability to suppress the polysulfide diffusion, a series of polysulfide penetration experiments are performed on H‐type cells (**Figure** **4**). The right chamber contains 0.25 mol L^−1^ Li_2_S_6_ (1,2‐dimethoxyethane (DME) as solvent) and the left chamber contains blank DME solvent. As expected, PP separator cannot suppress the diffusion of polysulfide species. Thus, the transparent solvent changes from colorless to yellow at the simultaneous time of injecting Li_2_S_6_ solution, indicating the diffusion of small amount of long‐chain Li_2_S_6_. After aging for 6 h, the color of left side completely changes to dark yellow, suggesting a large amount of polysulfides pass through the pristine PP separator. In stark contrast, no color change is observed in the H‐type cells with NCP‐CPE at the start. As time elapses, there occurs a little light yellow in the left chamber, implying the polysulfide migration is significantly suppressed by the introduction of NCP‐CPE. In addition, we also use the Li_2_S_4_ solution to examine the capability of NCP‐CPE to block polysulfide diffusion (displayed in Figure S9, Supporting Information). As expected, the Li_2_S_4_ cannot pass through the NCP‐CPE to the blank DME solvent. In contrast, the PP separator is incompetent to prevent the polysulfides from shuttling. Figure S10 (Supporting Information) exhibits the polysulfide absorption experiment. When a piece of NCP‐CPE is added to the polysulfide solution, the yellow solution turns into colorless one. These visible results reveal that the NCP‐CPE can effectively suppress the notorious shuttle effects. The polar functional groups such as hydroxyl in NCP‐CPE may bond with lithium polysulfides. Therefore, the diffusion of polysulfides can be effectively alleviated by employing NCP‐CPE.

Self‐discharge is a severe challenge brought about by the polysulfides shuttle, causing the loss of active materials and low Coulombic efficiency.^66^, ^67^ Figure S11b (Supporting Information) shows the initial discharge curve of Li‐S battery at 0.5 C with liquid electrolyte and NCP‐CPE after aging for 40 d. The battery with NCP‐CPE can still deliver a capacity of 865 mA h g^−1^, which is 95.3% of the fresh battery (Figure S11a, Supporting Information). In comparison, the battery with liquid electrolyte can only deliver a capacity of 640 mA h g^−1^ (only 79% of the fresh battery). These results demonstrate that NCP‐CPE is effective in reducing the migration of polysulfides from cathode side to the anode side. Figure S12a,b (Supporting Information) presents the cycle performance of Li‐S battery with NCP‐CPE and liquid electrolyte at 0.5 C after aging for 40 d, respectively. It is worth noting that the capacity of battery using NCP‐CPE drops to 760 mA h g^−1^ in the first three cycles which can be assigned to the activation process,^55^ Then, the capacity modestly falls to 647 mA h g^−1^ in the subsequent 200 cycles with a high Coulombic efficiency over 98% (Figure S13, Supporting Information). These results reveal that a highly anti‐self‐discharge ability has been achieved, which is because NCP‐CPE can effectively block and absorb the dissolved polysulfides.

To investigate the reason why NCP‐CPE can significantly improve the performance of Li‐S battery, the cycled battery was disassembled and measured. As shown in Figure S14a (Supporting Information), the digital image of cycled NCP‐CPE maintains good integrity. Figure S14b (Supporting Information) presents SEM image of cycled NCP‐CPE. Compared to the fresh NCP‐CPE, the surface coating on cellulose keeps intact after 1500 discharge/charge cycles, implying a robust structural integrity for the long‐term cycles.

**Figure 5:**
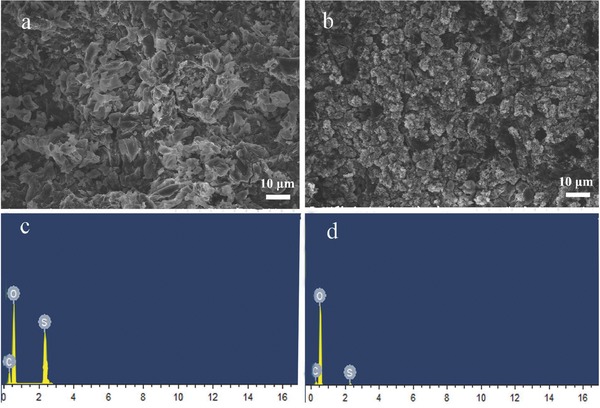
Typical SEM images and the corresponding EDS spectra of cycled Li anode with a,c) liquid electrolyte and b,d) NCP‐CPE.

**Figure** **5** displays the SEM images and corresponding energy‐dispersive spectrometer (EDS) results of cycled Li anode using liquid electrolyte (Figure 5a,c) and NCP‐CPE (Figure 5b,d). It is ubiquitous that the lithium dendrites grow and pulverize in lithium anode during the cycling process in Li metal‐based batteries. LiNO_3_ is an amazing additive to form a stable solid electrolyte interface (SEI) film with the assistance of polysulfides in Li‐S battery.^68^, ^69^, ^70^ However, the excessive shuttled polysulfides would inevitably react with Li anode and destroy the structure of SEI film. The side reaction leads to loss of active materials, which results in fast capacity decay and low Coulombic efficiency.^71^ As shown in Figure 5a, the SEM image of cycled Li metal surface with liquid electrolyte is quite rough. It is evidently found that some branch‐like Li dendrites grow on Li metal anode. It is exciting that the surface of Li metal with NCP‐CPE is more smooth and there rarely exists the lithium dendrite (Figure 5b), indicating that the polymer coating of NCP‐CPE in the anode side can enable homogeneous Li^+^ stripping/plating and thus effectively suppress the lithium dendrite growth. As exhibited in Figure 5c,d, the EDS results of batteries with liquid electrolyte give a higher sulfur signal than the batteries with NCP‐CPE. These results disclose that the shuttle effect can be greatly suppressed by the use of NCP‐CPE.

**Figure 6:**
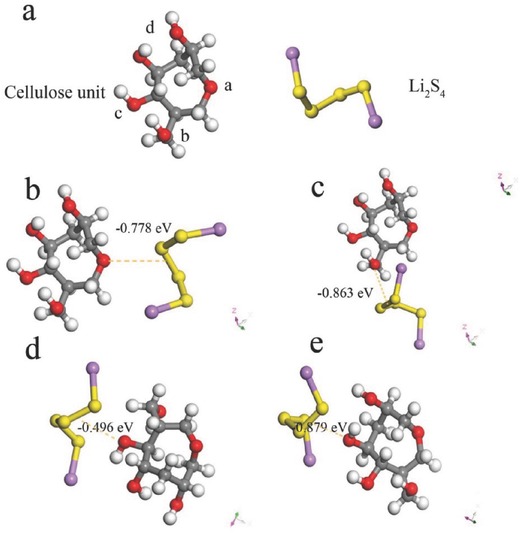
a) Geometrical structure of repeating unit in cellulose and Li_2_S_4_. Binding energy of Li_2_S_4_ to b) a site, c) b site, d) c site, and e) d site. Color scheme: red, gray, white, yellow, and purple spheres represent O, C, H, S, and Li atoms, respectively.

To better illustrate the interactions between polysulfides and oxygen‐containing groups in cellulose, density functional theory (DFT) calculation is performed to unveil the binding energy of polysulfide on cellulose. Here, we choose Li_2_S_4_ as the prototype for simulation calculation, for it plays an important role in the electrochemical interconversion of S_8_ and Li_2_S. **Figure** **6** shows the geometrical structure of Li_2_S_4_ and repeating units in cellulose. We choose four different “oxygen sites” in the cellulose (shown in Figure 6a), named as “a‐, b‐, c‐, d‐site.” The adsorption energy (*E*_d_) is calculated according to the equation: *E*_d_ = [*E*_total_ − *E*_Li2S4_ − *E*_a_], where *E*_total_ is the total energy of the Li_2_S_4_ adsorption sites on the cellulose, *E*_Li2S4_ and *E*_a_ are the total energies of Li_2_S_4_ and cellulose, respectively. And the more negative *E*_d_ indicates the stronger adsorption ability. The calculated *E*_d_ of different adsorption sites are listed in Table S2 (Supporting Information). It is apparent that all “O‐sites” show strong affinities with the Li_2_S_4_. The binding energies of Li_2_S_4_ on different “O‐sites (a,b,c,d)” are −0.778, 0.863, −0.496, and −0.879 eV, respectively. It is interesting that the binding energy of Li_2_S_4_ on d‐site is much stronger than the other sites. Actually, the binding mechanism between polysulfides and oxygen functional groups has been revealed in Zhang's work.^72^ He summarized the interaction between oxygen‐containing functional groups and polysulfides as follows: (1) O—S covalent bonds could be formed to immobilize the polysulfides, (2) the positively charged lithium atom in polysulfides can be electrostatically anchored by negatively charged oxygen via the Li bond (O—Li^+^), (3) the hydrogen bond formed between polysulfides and hydroxyl also has a beneficial effect on the suppression of the polysulfides immigration. These results indicate that the oxygen‐containing functional groups can strongly anchor the polysulfides, which will be further confirmed by the X‐ray photoelectron spectroscopy (XPS) study in the next part.

**Figure 7:**
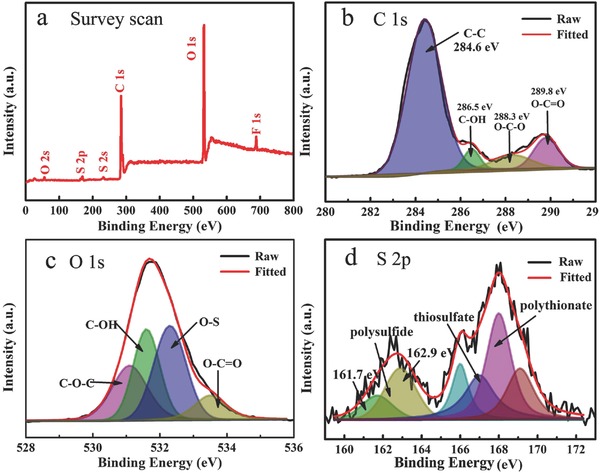
a) XPS survey spectra and high‐resolution XPS spectra of b) C 1s, c) O 1s, and d) S 2p.

XPS is used to detect the variation of elemental composition and functional groups in the cycled NCP‐CPE. According to the survey scan spectra of the cycled NCP‐CPE (**Figure** **7**a), C 1s, O 1s, S 2p, and F 1s can be clearly found in the full scan, which come from cellulose, polysulfides, and PVDF binder, respectively. These results confirm the fact that the polysulfides have been anchored on the cellulose backbone. Figure 7b displays the C 1s XPS spectrum of cycled NCP‐CPE. It comprises of peaks at 284.6, 286.5, and 288.3 eV, which are assigned to C—C, C—OH, and O—C—O in the cellulose,^73^ respectively. The peak at 289.8 eV corresponds to the C 1s core level of carbon atoms in carboxyl groups. It should be noted that there are no carboxyl groups in pure cellulose. According to the previous reported work, there are always some impurities in cellulose (mainly lignin).^44^ The new peak positioned at 289.8 eV should be assigned to the C=O in lignin. Figure 7c shows the high resolution O 1s spectra. Three peaks with positions at 531.1, 532.3, and 533.5 eV are corresponding to the species C=O, C—OH, and C—O—C, respectively. A new peak at 531.6 eV in the O 1s spectra can be ascribed to the O—S bond,^74^ which further confirms the immobilization of polysulfides on NCP‐CPE. From the S 2p spectra in Figure 7d, strong S 2p_3/2_ contributions at 161.7 eV (S_T_^−1^) and 162.9 eV (S_B_^−2^) with 1:2 intensity ratios are observed, suggesting a chain length approximately equal to Li_2_S_6_ which possesses the 2:4 atomic ratio of terminal/bridging sulfur in this polysulfide. S 2p peaks between 166 and 170 eV correspond to the formation of thiosulfate and polythionate, which can serve as highly effective polysulfide mediator to limit the diffusion of polysulfides.^75^, ^76^ The XPS results disclose the fact that the hydroxyl in NCP‐CPE has strong affinities with sulfur‐related active species, resulting in the enhanced battery performance.

**Figure 8:**
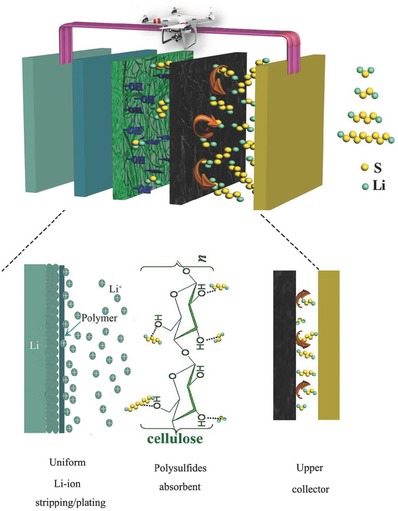
Schematic illustration of interaction mechanism between polysulfides and NCP‐CPE.

As mentioned above, the designed sandwich‐type polymer electrolyte can significantly enhance the comprehensive performance of Li‐S batteries including cyclability, rate capability, and lithium anode protection. This can be attributed to the unique structure of NCP‐CPE and synergistic effect of each functional layer. A schematic representation of multifunctional sandwich‐structured polymer electrolyte is displayed in **Figure** **8**. First, the nanocarbon coating toward cathode side may physically block the polysulfides diffusion path. It also functions as a second current collector, facilitating the electron transfer and providing more redox reaction sites to reactivate the active materials detached from the cathode. Second, the hydroxyls in cellulose nonwoven have strong affinities with polysulfides, which can effectively suppress the polysulfides shuttle. Third, the use of PEG‐PPG‐PEG coating layer in anode side can enable uniform lithium‐ion stripping/plating that gives a well protection of Li metal anode. As a result, the Li‐S batteries with such designed sandwich‐structured polymer electrolyte have achieved a leap forward in cycle stability and rate performance.

## 3 Conclusions

In summary, we have designed a novel sandwich‐structured polymer electrolyte (NCP‐CPE) to address the shuttle effect that severely hindered the practical application of Li‐S battery. Notably, Li‐S battery based on this multifunctional polymer electrolyte exhibits superior cycle performance for 1500 cycles and excellent rate capability up to 4 C with a pure sulfur cathode, which is comparable to the best reported value so far. On the cathode side, the nanocarbon coating can function as a second current collector, which facilitates the electron transfer and accelerates the conversion of polysulfides. In addition, the nanocarbon coating also employs as a physical barrier to mitigate the migration of polysulfides. Furthermore, the hydroxyl groups in cellulose backbone of NCP‐CPE have strong affinities with polysulfides which can chemically suppress the immigration of polysulfides. Finally, on anode side, PEG‐PPG‐PEG coating layer of NCP‐CPE can enable uniform lithium ion stripping/plating, which gives a well protection of Li metal anode. The demonstrated approaches here may guide us to go ahead to develop multifunctional polymer electrolyte for high‐performance Li‐S battery.

## 4 Experimental Section

*Preparation of Sulfur Cathode*: Sulfur cathode with a content of 60% was prepared by grinding commercial sulfur powder, Super P, and PVDF at a ratio of 6:3:1 in N‐methyl‐2‐pyrrolidone (NMP) solvent to form a uniform slurry. Then, the slurry was casted on an aluminum foil and followed by drying at 60 °C before use. Cathodes with sulfur contents of 70 and 80% were prepared with the same method. The average loading of sulfur in cathode was ≈1.5 4.9 mg cm^−2^.

*Synthesis of NCP‐CPE*: In preparation of NCP‐CPE, the cellulose nonwoven membrane was synthesized according to a previous study.^31^ Then, a facile traditional doctor‐blading method was used. Nanocarbon black was mixed with PVDF binder at a ratio of 4:1 in NMP solvent to form uniform slurry and then the slurry was coated on the cellulose. For preparing the (*M*_w_ = 5800) coating, some amount of PEG‐PPG‐PEG was dissolved in acetonitrile to form uniform solution. Then the transparent solution was casted on the opposite side of carbon‐coated cellulose nonwoven. The prepared NCP‐CPE was dried at 120 °C in a vacuum oven for 24 h and then transferred into argon‐filled glove box.

*Preparation of Li_2_S_6_ Solution*: To prepare the Li_2_S_6_ solution, sublimate sulfur and lithium sulfide were weighted at a stoichiometric mole of 5:1 and transferred into a 20 mL vial in a glove box. Then, the mixture was sealed and heated at 80 °C with vigorously stirring for 6 h. The as‐prepared Li_2_S_6_ solution was stored in the glove box before use.

*Characterization*: The SEM and EDS experiments were carried out with a field emission scanning electron microscope named Hitachi S‐4800. XPS analysis was performed on a Perkin‐Elmer PHI 550 spectrometer with Al Kα (1486.6 eV) as the X‐ray source. The cycled NCP‐CPE separator was first washed with DME solvent thoroughly for several times in the glove box before XPS measurement. The carbon C1s line with position at 284.6 eV was used as a reference for calibration.

*Electrochemical Performance*: The ionic conductivity of liquid electrolyte and NCP‐CPE was calculated from electrochemical impedance spectroscopy (EIS) using Biologic VMP‐300 potentiostat. The ionic conductivity of liquid electrolyte and the NCP‐CPE were analyzed by AC impedance method and calculated according to the formula: σ = *d*/(*s* × *R*), where σ is the ionic conductivity, *d* is the thickness of PP and NCP‐CPE, *s* is the surface area of the membrane, and *R* refers to the bulk resistance. CR2032 coin cells, consisting of a sulfur cathode, a PP or NCP‐CPE membrane, and a metallic Li anode, were fabricated in an argon‐filled glove box. The electrolyte was 1 m lithium bis(trifluoromethanesulfonyl) imide (LiTFSI) and 1 wt% lithium nitrate (LiNO_3_) in 1,3‐dioxolane (DOL) and DME (1:1 by volume) and around 50 µL electrolyte was added into each battery. CV was obtained with a Biologic VMP‐300 potentiostat at a scan rate of 0.1 mV s ^−1^ in the range of 1.7–2.8 V. The galvanostatic discharge and charge tests were performed with LAND battery‐test system in the range of 1.8–2.8 V.

*Computational Method*: All the computations in the calculation used in this study, including the geometry optimizations, were performed at the B3LYP/6‐311+G*(d, p) level of theory based on the DFT method. And a spin‐unrestricted scheme was used for the electron systems during structure optimized. In addition, the adsorption energies were given as following: *E*_d_ = [*E*_total_ − *E*_Li2S4_ − *E*_a_], where *E*_total_ was the total energy of the Li_2_S_4_ adsorption sites on the cellulose, *E*_Li2S4_ and *E*_a_ was the total energy of Li_2_S_4_ and cellulose, respectively.

## Conflict of Interest

The authors declare no conflict of interest.

## Supporting information

Supplementary
