# Supplementary material for: Multifunctional Sandwich‐Structured Electrolyte for High‐Performance Lithium–Sulfur Batteries
Source: Adv Sci (Weinh). 2018 Jan 2;5(3):1700503. doi: 10.1002/advs.201700503 (PMC5867043; doi:10.1002/advs.201700503)
Supplement: Supplementary file 1 — Supplementary [file ADVS-5-1700503-s001.pdf]

Copyright WILEY-VCH Verlag GmbH & Co. KGaA, 69469 Weinheim, Germany, 2017.

ADVANCED SCIENCE
Open Access

Supporting Information

for *Adv. Sci.,* DOI: 10.1002/advs.201700503

Multifunctional Sandwich-Structured Electrolyte for High-Performance Lithium–Sulfur Batteries

*Hongtao Qu, Jianjun Zhang, Aobing Du, Bingbing Chen, Jingchao Chai, Nan Xue, Longlong Wang, Lixin Qiao, Chen Wang, Xiao Zang, Jinfeng Yang, Xiaogang Wang, and Guanglei Cui**

WILEY-VCH

Copyright WILEY-VCH Verlag GmbH & Co. KGaA, 69469 Weinheim, Germany, 2017.

Supporting Information

Multifunctional Sandwich-structured Electrolyte for High Performance Lithium-Sulfur Batteries

Hongtao Qu‡*, Jianjun Zhang*‡*, Aobing Du, Bingbing Chen, Jingchao Chai, Nan Xue, Longlong Wang, Lixin Qiao, Chen Wang, Xiao Zang, Jinfeng Yang, Xiaogang Wang, Guanglei Cui**

‡ These authors contribute equally to this work.

H. T. Qu, J. J. Zhang, A. B. Du, B. B. Chen, J. C. Chai, N. Xue, L. L. Wang, L. X. Qiao, C. Wang, X. Zang, J. F. Yang, Prof. X. G. Wang, Prof. G. L. Cui

Qingdao Industrial Energy Storage Technology Institute

Qingdao Institute of Bioenergy and Bioprocess Technology, Chinese Academy of Sciences

Qingdao 266101, P. R. China.

E-mail: cuigl@qibebt.ac.cn

H. T. Qu, J. J. Zhang, A. B. Du, J. C. Chai, N. Xue, L. L. Wang, C. Wang, J. F. Yang,

University of Chinese Academy of Sciences, No.19A Yuquan Road, Beijing, China, 100049

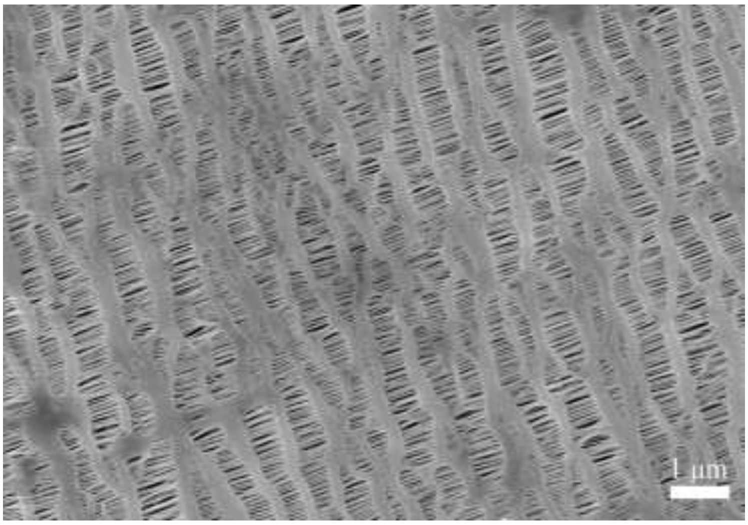

Figure S1. Typical SEM image of PP separator.

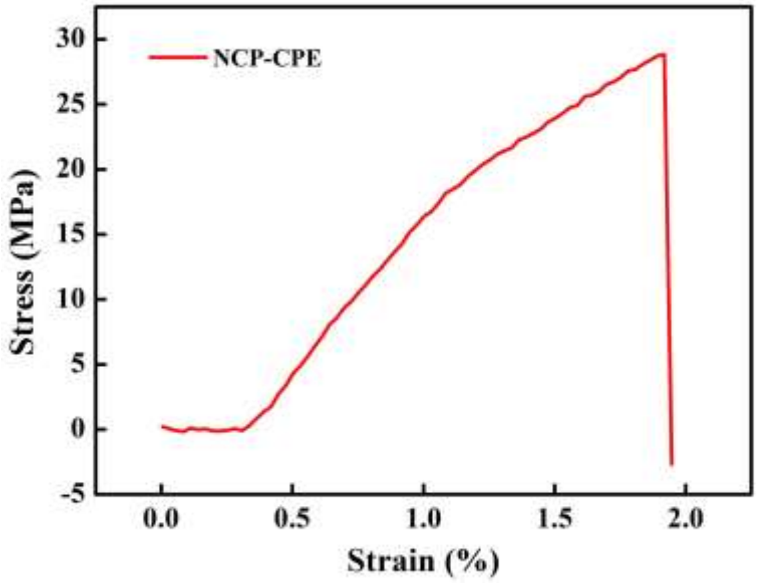

Figure S2. Stress-strain curve of NCP-CPE.

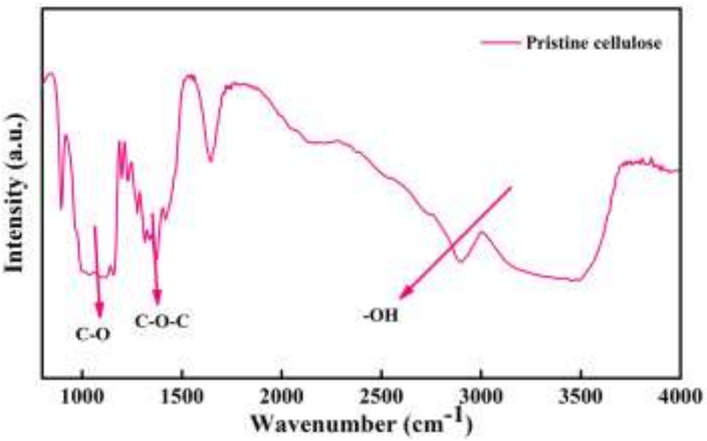

Figure S3. FTIR spectroscopy of cellulose nonwoven.

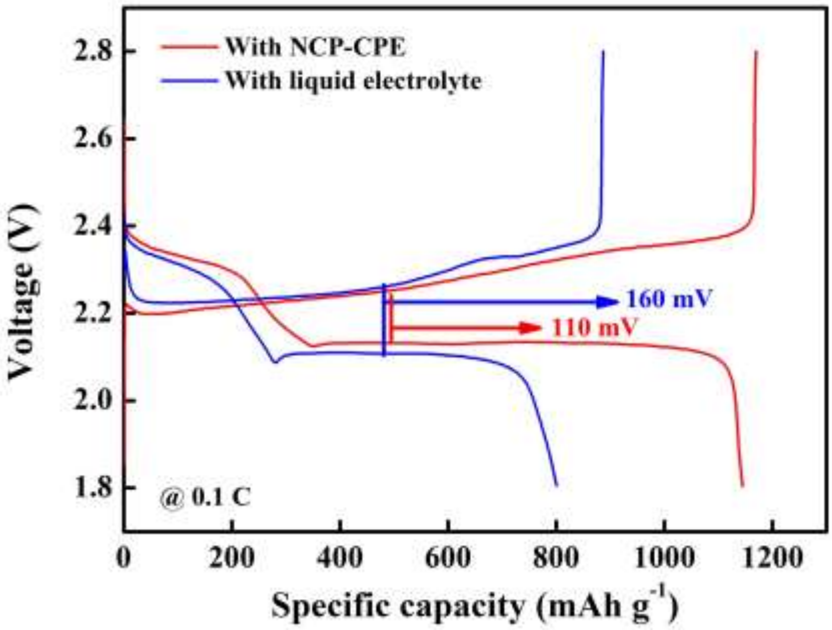

Figure S4. The charge/discharge profiles of Li-S batteries with liquid electrolyte and NCP-CPE at 0.1 C.

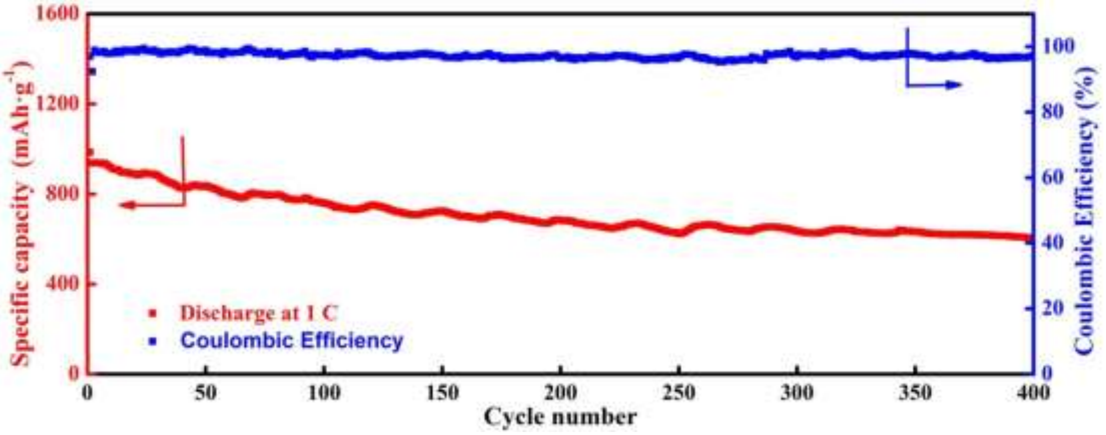

Figure S5. Cycle performance of Li-S battery (60 % sulfur content) with NCP-CPE at 1 C.

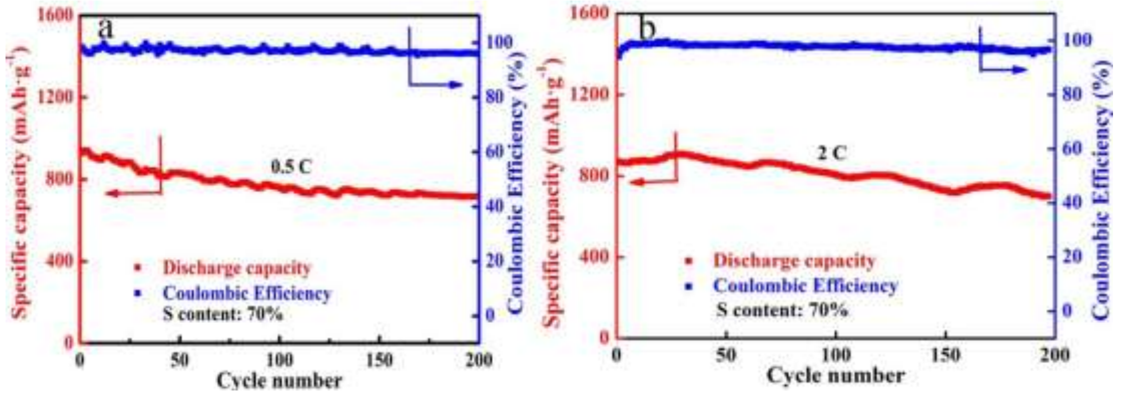

Figure S6. Cycling stability of Li-S battery (70 % sulfur content) with NCP-CPE at a) 0.5 C and b) 1 C.

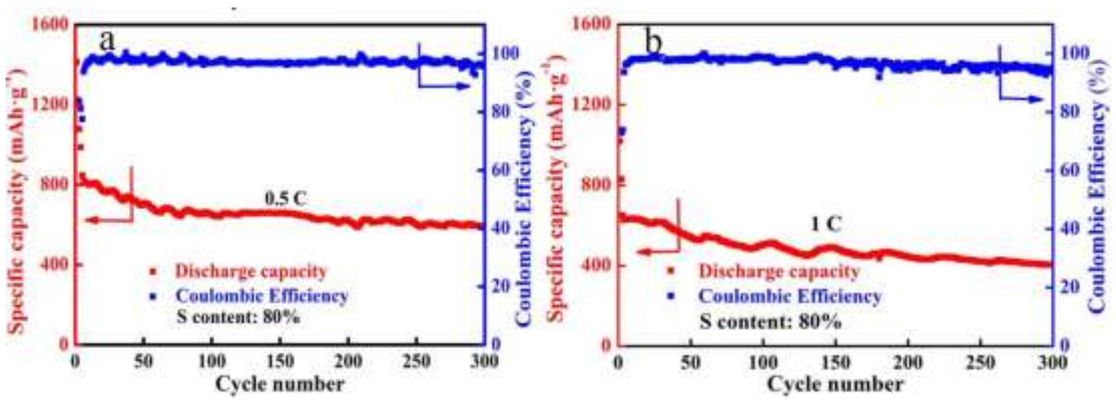

Figure S7. Cycling stability of Li-S battery (80 % sulfur content) with NCP-CPE at a) 0.5 C and b) 1 C.

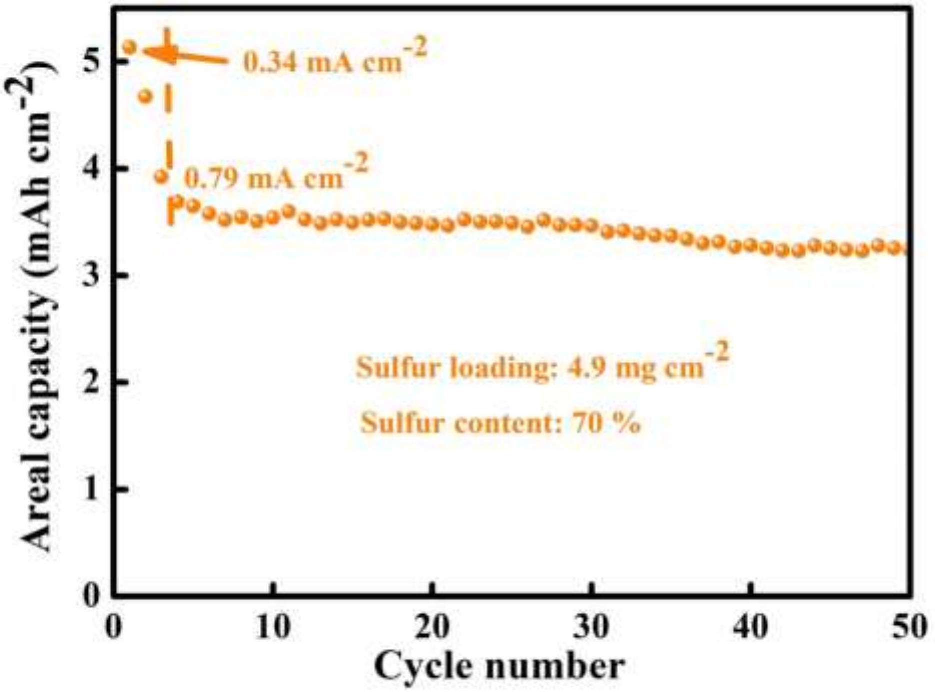

Figure S8. Cycle performance of high sulfur loaded (4.9 mg cm^{-2}) Li-S battery with NCP-CPE.

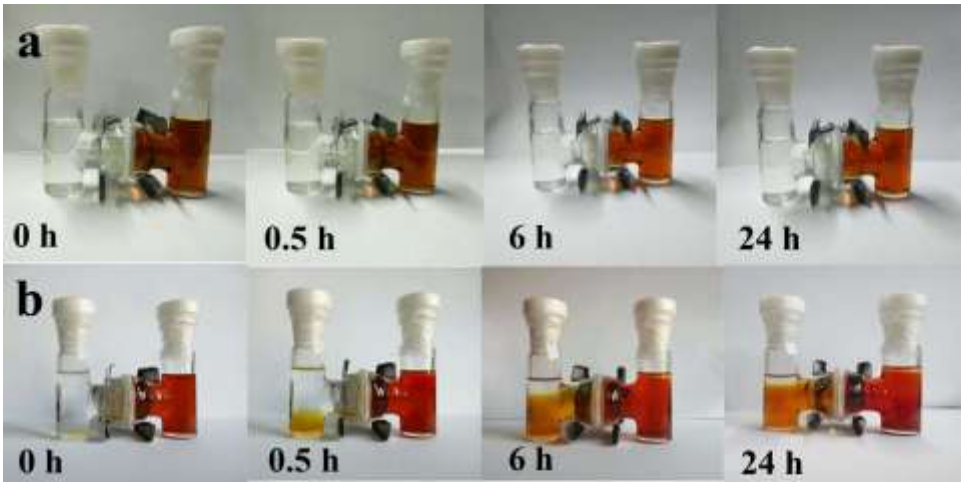

Figure S9. Digital photos of H-type cells separated by NCP-CPE a) and PP separator b).

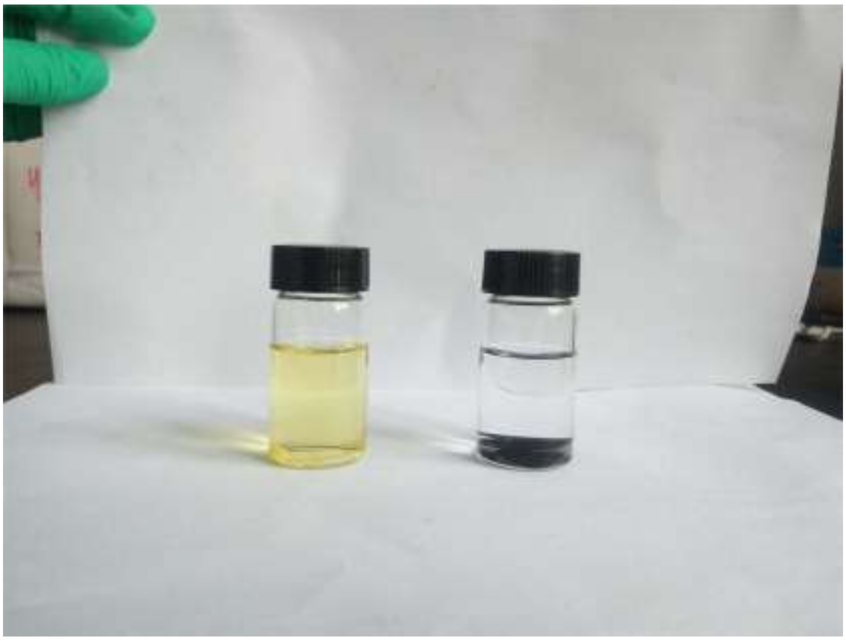

Figure S10. Digital photo of polysulfide adsorption test (50 mM Li_2S_6 dissolved in DME).

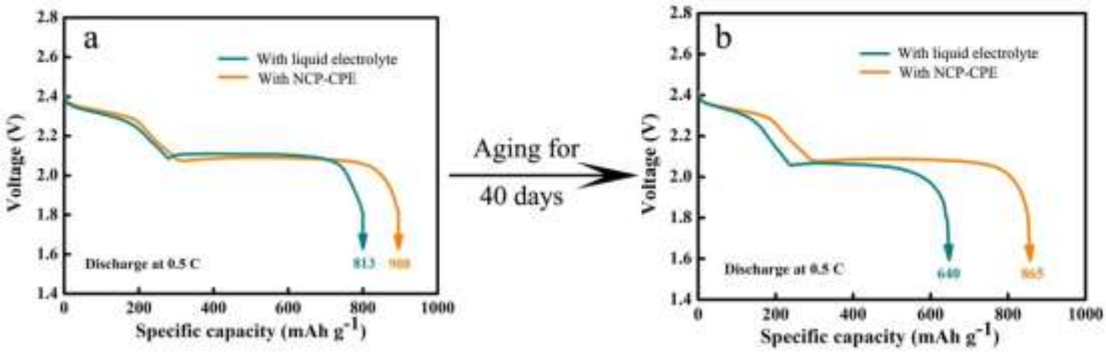

Figure S11. The 1st discharge profiles of Li-S battery at 0.5 C: a) fresh cell, b) aging for 40 days. (Fresh cell: The newly assembled Li-S battery was aged for 4 hrs to ensure the cathode completely infiltrated and then discharged at 0.5 C. For anti-self-discharge measurement, Li-S battery was also assembled and stored for 40 days, then discharged at 0.5 C).

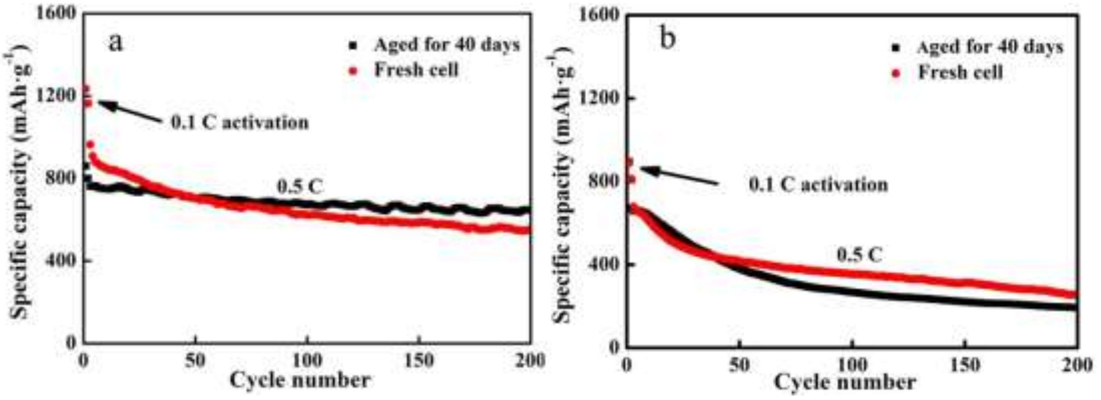

Figure S12. Comparison of cycle performance of Li-S battery before/after aging for 40 days with a) NCP-CPE and b) liquid electrolyte.

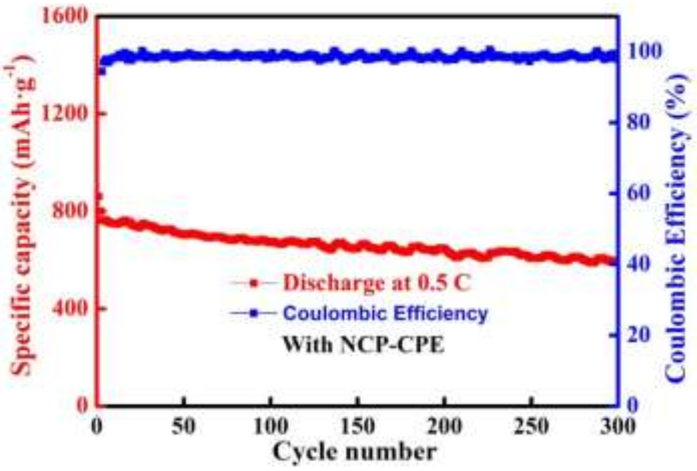

Figure S13. Cycle performance of Li-S battery with NCP-CPE after aging for 40 days.

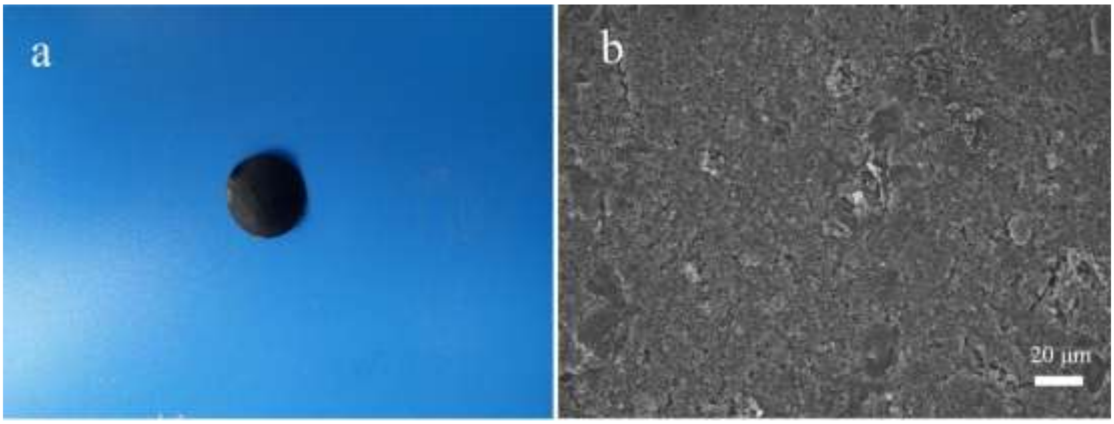

Figure S14. a) Digital photo and b) typical SEM image of NCP-CPE which was disassembled from Li-S cell after 1100 cycles.

Table S1. Electrochemical performance comparisons of previously reported Li-S battery and our presented case.

Separator/ Polymer electrolyte	Cathode	S content/ wt %	Cycle performance			Rate performance		Refs
			Cycle number	C-rate	Capacity decay per cycle / %	C-rate	Reversible capacity / mAh·g^{-1}	
PP/Ketjen black	KB/S	64	100	1	0.14	2	900	[49]
PP/Super P	Bare S	60	100	0.5	0.28	2	700	[50]
PP/MC	Bare S	70	500	0.5	0.081	2	850	[51]
PP/Super P/Nafion	Bare S	70	200	0.5	0.2	1	302	[52]
PP/AB-SO_3^-	C-PANI/S	72	100	0.1	0.24	1.5	800	[43]
PP/MoS_2	Bare S	65	600	0.5	0.083	1	500	[53]
PP/Black phosphorus	Bare S	80	100	0.2	0.14	2	600	[54]
GO/MOF	CMK-3/S	48	1500	1	0.019	3	500	[55]
PP/RGO	Bare S	70	100	0.2C	0.18	2	710	[56]
PP/$Li_3Ti_5O_{12}$/Graphene	Bare S	70	500	1	0.0286	2	700	[57]
PP-AB&CNT&LAGP	C/S	56	150	0.5	0.224	1	852	[58]

PETEA	Bare S	60	400	0.5	0.047	1	601.2	[28]
PP/$BaTO_3$	Bare S	60	50	0.1	0.34	~	~	[59]
Py_{14}TFSI/DOL/DME	KB/S	64	120	0.1	0.185			[60]
PVDF-HFP	KB/S	53	40	0.3	0.5			[61]
PVDF-HFP/ PMI_mTFSI	Bare S	60	30	0.1	0.49	1	311	[29]
Nafion	S catholyte		500	0.5	0.11	0.5	900	[62]
PAN/GO	Bare S	70	100	0.2	0.395	2	337	[63]
**NCP-CPE**	**Bare S**	**60**	**1500/400**	**0.5/1**	**0.039/0.047**	**4**	**594**	**Our work**
		**70**	**200/200**	**0.5/2**	**0.106/0.097**	**2**	**871**	
		**80**	**300/300**	**0.5/1**	**0.104/0.119**	**2**	**441**	

Table S2. The binding energy of Li_2S_4 to different oxygen sites in cellulose.

Oxygen site	a	b	c	d
Binding Energy (eV)	-0.778	-0.863	-0.496	-0.879
